# Indikative Präventionsprogramme zur Förderung der seelischen Gesundheit im Vor- und Grundschulalter: Teilnahmebereitschaft von Kinderärzt*innen und Familien an einer innovativen Versorgungskette

**DOI:** 10.1007/s00103-023-03787-0

**Published:** 2023-11-03

**Authors:** Max Weniger, Katja Beesdo-Baum, Julia Ernst, Cornelia Beate Siegmund, Patricia Theresa Porst, Maria McDonald, Veit Roessner, Susanne Knappe

**Affiliations:** 1 Institut für Klinische Psychologie und Psychotherapie, Professur für Behaviorale Epidemiologie, Technische Universität Dresden, Chemnitzer Straße 46, 01187 Dresden, Sachsen Deutschland; 2 Klinik und Poliklinik für Kinder- und Jugendpsychiatrie und -psychotherapie, Universitätsklinikum und Medizinische Fakultät Carl Gustav Carus, Technische Universität Dresden, Dresden, Sachsen Deutschland; 3 Evangelische Hochschule Dresden, Dresden, Sachsen Deutschland

**Keywords:** Emotionale und Verhaltensprobleme im Kindesalter, Prävention, Regelversorgung, Screening psychischer Auffälligkeiten, Strengths and Difficulties Questionnaire (SDQ), Childhood emotional and behavioral problems, Prevention, Routine care, Screening for mental health problems, Strengths and Difficulties Questionnaire (SDQ)

## Abstract

**Hintergrund:**

Psychische Auffälligkeiten beginnen häufig im Kindesalter und können in psychische Störungen münden. Vorhandene Präventionsangebote werden trotz Wirksamkeit nur spärlich in Anspruch genommen. Ziel war zu prüfen, inwiefern durch die Etablierung einer Versorgungskette Risikokinder frühzeitig identifiziert und Präventionsmaßnahmen zugewiesen werden können, sowie inwieweit indikative Präventionsprogramme schlussendlich in Anspruch genommen werden.

**Methoden:**

In einer prospektiven Implementationsstudie wurde während der regulären U9- bis U11-Gesundheitsuntersuchungen (Altersbereich: 5–10 Jahre) der „Strengths and Difficulties Questionnaire“ als Screeninginstrument an Familien ausgegeben. Diese erhielten von ihren Kinderärzt*innen unmittelbar eine Ergebnisrückmeldung und im Falle von grenzwertig auffälligen emotionalen oder Verhaltensproblemen eine Empfehlung für ein indikatives Präventionsprogramm. Vor Programmteilnahme fand im Vorgespräch eine Indikationsprüfung statt.

**Ergebnisse:**

Im Raum Dresden beteiligten sich *n* = 46 (38,7 %) Kinderärzt*innen am Projekt. In *n* = 28 Kinderarztpraxen nahmen *n* = 3231 (86,4 %) Familien am Screening teil, *n* = 864 (26,7 %) Kinder, deren Familien eine Ergebnisrückmeldung erhielten, bekamen eine Präventionsempfehlung. Zur Präventionsprogrammteilnahme meldeten sich *n* = 118/864 (13,7 %) Familien selbstständig. *n* = 215/624 (35,5 %) zeigten Interesse nach projektinitiierter Kontaktaufnahme. Über andere Zugangswege kamen *n* = 139 Teilnahmeanfragen. *n* = 337 *(n* *=* *461; über alle Zugangswege) *Vorgespräche wurden geführt. Schließlich nahmen *n* = 237 *(n* *=* *337) *Kinder ein indikatives Präventionsprogramm in Anspruch.

**Schlussfolgerung:**

Eine Ausweitung der Vorsorgeuntersuchung auf psychische Auffälligkeiten ist umsetzbar, nützlich und erfährt breite Akzeptanz. Um eine Versorgungskette einzurichten, sollte eine Angebotsstruktur etabliert werden, um damit die Zuweisung zu und Inanspruchnahme von Präventionsmaßnahmen zu ermöglichen.

## Abstract (en)

**Background:**

Mental health problems usually have their onset in childhood. Undiagnosed, they may progress into mental disorders. Despite their effectiveness, existing preventive programs have been rarely used. We aimed to examine to what extent the establishment of a care chain can identify children at high risk at an early stage and assign them to preventive interventions. In addition, prevention program participation was assessed.

**Methods:**

In a prospective implementation study, the Strengths and Difficulties Questionnaire was administered as a screening instrument to families during regular pediatric health examinations (U9–U11, child age 5–10 years). Families received feedback directly from the pediatrician, and in the case of borderline abnormal emotional or behavioral problems, a recommendation for an indicative prevention program. Program indication was additionally determined in an entry examination prior to program participation.

**Results:**

In the area of Dresden (Germany), *n* = 46 (38.7%) pediatricians participated in the project. In *n* = 28 pediatric practices, *n* = 3231 (86.4%) families participated in the screening and *n* = 864 (26.7%) children received a prevention recommendation. Of the families, *n* = 118/864 (13.7%) self-registered for the prevention programs, *n* = 215/624 (35.5%) showed interest after being contacted by the study teamn. Through other pathways, *n* = 139 families requested participation. Clinical evaluation interviews to assess prevention indication were conducted in *n* = 337 children (*n* = 461; via all entry pathways). Finally, *n* = 237 (*n* = 337) children participated in an indicated prevention program.

**Conclusion:**

Expanding screening to mental health problems during regular health checkups is feasible, useful, and widely accepted. In order to implement a care chain, a supply structure should be established to enable referral to and uptake of preventive interventions.

## Einleitung

Zur Förderung von Prävention wurde 2015 in Deutschland das Präventionsgesetz [1, 2] verabschiedet. Primärziel ist die Vermeidung von Krankheiten, bevor sie entstehen – der Fokus wird erweitert von vornehmlich kurativen hin zu (primär-)präventiven Maßnahmen. Dafür sollen Rahmenbedingungen geschaffen werden, die Versorgungsstrukturen und -angebote zur Prävention stärken. In der Folge haben sich die Leistungsausgaben für „Prävention in Lebenswelten“, „Betriebliche Gesundheitsförderung“ und „Individuelle verhaltensbezogene Prävention“ von 4,49 € (2015) auf 8,64 € (2019) je Versicherten/Jahr beinahe verdoppelt. Insgesamt wurden 2019 für individuelle verhaltensbezogene Präventionsmaßnahmen, die letztgenanntem Bereich zugeordnet werden, im Handlungsfeld Stressmanagement ca. 65 Mio. € von den gesetzlichen Krankenversicherungen aufgewendet [3].

Ein Großteil psychischer Störungen bleibt trotz ihres oftmals frühen Beginns im Kindes- und Jugendalter unerkannt und damit unbehandelt [4–6]. Umso mehr ist anzunehmen, dass psychische Auffälligkeiten unterhalb der diagnostischen Schwelle nicht erkannt werden, wodurch die Optionen für Präventivmaßnahmen erheblich geschmälert sind. Psychische Auffälligkeiten sind als Risikofaktor für die Entwicklung von klinisch relevanten psychischen Störungen belegt [7]. Diese gehen meist mit erheblichen individuellen Belastungen einher [8] und bilden die zweitteuerste Erkrankungsgruppe in Deutschland [9]. Allein die direkten Krankheitskosten wurden im Jahr 2015 auf 44 Mrd. € beziffert; davon entfallen über 3 Mrd. € auf unter 15-Jährige [10]. Dies verdeutlicht den gegenwärtig geringen, aber zunehmenden Stellenwert der Prävention psychischer Störungen.

Der derzeitige Zugangsweg für Kinder in die Gesundheitsversorgung erfolgt überwiegend über niedergelassene Fachkräfte der Kinder- und Jugendmedizin (FfPaed) und der Allgemeinmedizin (FfAM). Derzeit nehmen etwa 85 % (Jahresprävalenz) der 3‑ bis 10-jährigen Kinder pädiatrische Leistungen in Anspruch [11]. Auch bei Hinweisen auf psychische Auffälligkeiten konsultiert der Großteil der Familien (87 %) FfPaed/FfAM, nur 29 % nehmen psychiatrisch-psychotherapeutische Versorgungsangebote in Anspruch [5]. Krankenkassengeförderte Präventionsangebote zur individuellen Verhaltensprävention im Handlungsfeld Stressmanagement (29 %) wurden über alle Altersgruppen hinweg nach dem Bereich Bewegung (68 %) am zweithäufigsten in Anspruch genommen. Allerdings wurden diese von unter 20-Jährigen im Vergleich zu allen anderen Alterskohorten mit Abstand am seltensten genutzt [3]; die Zugangswege in die Prävention sind dabei kaum erforscht.

Die frühzeitige Erkennung und Behandlung psychischer Auffälligkeiten können sich positiv auf deren weiteren Verlauf und Prognose auswirken [12, 13] und zudem volkswirtschaftliche und individuelle Kosten reduzieren [8–10]. Daher sind eine Optimierung der Früherkennung und die Bereitstellung verhaltenspräventiver Angebote zentral, um deren Inanspruchnahme zu erhöhen. Ein Ansatz zur frühzeitigen Identifikation psychischer Auffälligkeiten bei Kindern kann der Einsatz eines Screeninginstrumentes sein [14, 15]. Solche werden derzeit jedoch nicht regelhaft, sondern auf Wunsch der Eltern oder in Verdachtsfällen von der FfPaed eingesetzt. Da FfPaed eine „Gatekeeper“-Funktion in der Gesundheitsversorgung von Familien obliegt und dort reguläre Vorsorgeuntersuchungen von 98,1 % der Familien, unabhängig von Migrationshintergrund und sozioökonomischem Status, wahrgenommen werden [16], bieten sich Kinderarztpraxen als Einrichtungen für ein Screening an. Somit könnten auch Familien mit einem höheren Präventionsbedarf angesprochen werden, welche möglicherweise aufgrund eines geringeren sozioökonomischen und Bildungshintergrundes zuvor nur schwer erreicht wurden („Präventionsdilemma“ [17]).

Ziel der vorliegenden regionalen prospektiven Implementationsstudie [18] war die Erprobung einer spezifischen Versorgungskette: vom Einsatz eines Screeninginstrumentes zur Identifikation psychischer Auffälligkeiten im Rahmen der regulären Vorsorgeuntersuchungen (U-Untersuchungen, U9: 5–6 Jahre, U10: 7–8 Jahre, U11: 9–10 Jahre) über die Ergebnisrückmeldung und ggf. Präventionsempfehlung seitens der FfPaed bis zur Inanspruchnahme eines indikativen Präventionsprogrammes. Untersucht wurden die Teilnahmebereitschaft der angefragten FfPaed und Familien am Screening, Zugangswege in die Versorgung und die Teilnahme an indikativen Präventionsprogrammen. Ablehnungsgründe im Laufe der Versorgungskette wurden ebenfalls erfasst.

## Methoden

### Studiendesign und Untersuchungsablauf

Das PROMPt-Projekt (Primärindikative und optimierte Zuweisung zu gezielten Maßnahmen bei emotionalen und Verhaltensauffälligkeiten bei Kindern) wurde von 11/2018 bis 09/2022 in Dresden und Umland durchgeführt. Detaillierte Informationen zur Methodik wurden bereits berichtet [18].

Für die Umsetzung des Projektvorhabens wurden im Zeitraum 01/2020 bis 06/2021 alle in der Kassenärztlichen Vereinigung Sachsen gelisteten niedergelassenen FfPaed in Dresden und im Umkreis von 20 km postalisch kontaktiert. Sie erhielten ein Anschreiben, Informationen über das Projekt sowie einen Antwortbogen. Dieser erfasste bei Studieninteresse Kontaktmöglichkeiten und bei Nichtteilnahme Ablehnungsgründe. Weiterhin wurde bei Ablehnung erfragt, ob die FfPaed bereit wäre, einen anonymisierten Arztfragebogen auszufüllen und/oder Informationsflyer zum Projekt in der Praxis auszulegen. Bei Interesse wurde ein Einweisungstermin mit der FfPaed und deren Praxispersonal vereinbart. Falls die FfPaed keine Rückmeldung gab, wurde nach 2 Wochen ein Erinnerungsbrief versendet. Wenn erneut keine Rückmeldung einging, erfolgten max. 5 telefonische Kontaktversuche.

**Figure 1:**
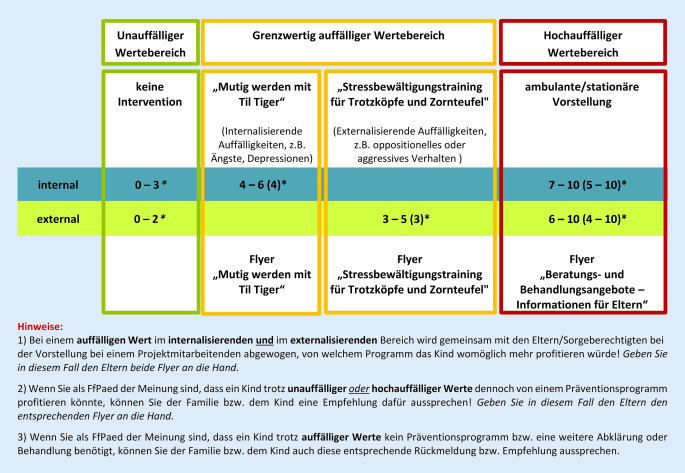

Familien, die zu einer U9- bis U11-Untersuchung in eine teilnehmende Kinderarztpraxis kamen, erhielten im Zeitraum von 02/2020 bis 09/2021 durch das in die Studie eingewiesene Praxisteam eine Mappe mit einer Studieninformation, einer Einwilligungserklärung (EWE) für Datenverarbeitung und Kontaktaufnahme, einem Screeningfragebogen zu Stärken und Schwächen ihres Kindes (SDQ: Strengths and Difficulties Questionnaire; [19]) und einem Fragebogenheft. Praxismitarbeitende werteten den SDQ unmittelbar mithilfe einer Schablone aus. Die ursprünglichen Cut-off-Werte [19] wurden für die vorliegende Studie modifiziert (siehe Abschnitt „Erhebungsinstrumente“). Eine tabellarische Einordnung der Werte (unauffällig/grenzwertig auffällig/hochauffällig) diente als Orientierungshilfe für die Empfehlung der FfPaed (Abb. 1). Letztere sprachen unter Berücksichtigung ihrer Expertise eine Empfehlung aus (kein Handlungsbedarf/Prävention/klinische Abklärung bzw. Behandlung), dokumentierten diese auf einem Kurzfragebogen (Arztbeurteilung) und übergaben ggf. einen Flyer für weiterführende Maßnahmen (Ansprechpartner/Hilfsangebote/Anlaufstellen). Alle in der Praxis verbliebenen Unterlagen (SDQ, EWE, Arztbeurteilung, Fragebogenheft) wurden dem Studienteam postalisch zugesandt.

Im Falle grenzwertig auffälliger Werte auf den SDQ-Subskalen „Emotionale Probleme“ und/oder „Verhaltensprobleme“ erhielten die Familien eine Empfehlung für die Teilnahme an einem innerhalb der Studie angebotenen indikativen Präventionsprogramm ([20, 21]; Abb. 1). Beide Programme sind nach § 20 Abs. 1 SGB V als theorie- und evidenzbasierte Frühpräventionsmaßnahmen mit kognitiv-behavioraler Ausrichtung zum multimodalen Stressmanagement anerkannt und somit Teil der Regelversorgung. Familien mit Interesse daran wurden gebeten, mit dem Studienteam Kontakt aufzunehmen. Anschließend erfolgte mit einem Studienmitarbeitenden (M. Sc. Psycholog*in) ein 50-minütiges sog. Vorgespräch zur Indikationsprüfung sowie zur Aufklärung über Inhalt und Rahmenbedingungen (u. a. Anzahl zertifizierter Trainer*innen bzw. Kinder je Gruppe; Sitzungsintervall) des empfohlenen Programmes und der begleitenden Fragebogenerhebungen. Sofern sich die Familien innerhalb von 3–4 Wochen nach der Präventionsempfehlung nicht selbstständig beim Studienteam meldeten und eine Einwilligung zur Kontaktaufnahme vorlag, erfolgten max. 5 Kontaktversuche per Telefon, 3 via E‑Mail und/oder 2 per Post im Abstand einer Woche (E-Mail) bzw. von 10 Tagen (Brief).

Ausschlusskriterien für die Teilnahme am Screening in der Kinderarztpraxis waren unzureichende Deutschkenntnisse/Lesefertigkeiten beim Elternteil sowie ein privat versichertes Kind. Für die Teilnahme am Vorgespräch/Präventionsprogramm waren Ausschlussgründe das Vorliegen einer aktuellen Diagnose (≤ 6 Monate) nach ICD-10 im Spektrum internalisierender/externalisierender Störungen, eine aktuelle psychotherapeutische Behandlung, instabile Medikation oder akute Selbst‑/Fremdgefährdung und unzureichende Sprachkenntnisse.

### Erhebungsinstrumente

Mittels projektspezifischer Fragebögen wurden Angaben zu den FfPaed und den Familien sowie Ablehnungsgründe erhoben. Zur Beschreibung der FfPaed wurden Angaben zum Alter, Geschlecht und der niedergelassenen Berufserfahrung in Jahren mittels Arztfragebogen erfragt. Ergänzend wurden Angaben zu den Patient*innen (Zeit in Minuten pro Patient*in, Anzahl Patient*innen pro Tag, Anzahl U‑Untersuchungen pro Quartal, Zeiten ohne U‑Untersuchungen, Anteil Patient*innen mit Migrationshintergrund) und zur Praxis (Anzahl Mitarbeiter*innen) eingeholt. Zusätzlich schätzten die FfPaed ihre Kompetenz hinsichtlich des Erkennens von psychischen Auffälligkeiten bei Kindern ein und gaben Auskunft darüber, ob ihnen Präventionsprogramme bekannt sind. Weiterhin machten sie Angaben über den bisherigen Einsatz eines Fragebogens zur Erhebung psychischer Auffälligkeiten und die Bekanntheit sowie den Einsatz von Muster 36 (Formular für eine Präventionsempfehlung). Zudem wurden die FfPaed befragt, wie häufig sie in den letzten 2 Jahren Weiterbildungsveranstaltungen zum Thema „psychische Gesundheit bei Kindern“ besucht haben und wie sie verfahren, wenn sie bei einem/einer ihrer Patient*innen erstmals psychische Auffälligkeiten beobachten. Eine qualitative Erhebung zur Zufriedenheit mit dem eingesetzten SDQ und dem Studienprozedere erfolgte unter *n* = 4 FfPaed, *n* = 4 Praxispersonal und *n* = 17 Familien [22].

Auf dem Arztbeurteilungsfragebogen wurden die FfPaed gebeten, nach ihrer Ergebnisrückmeldung an die Familien 4 Items auszufüllen. Dabei sollten sie angeben, welche Empfehlung bzw. Einschätzung sie nach dem SDQ-Screening ausgesprochen haben (keine Intervention; „Mutig werden mit Til Tiger“ [20]; „Ein Stressbewältigungstraining für Trotzköpfe und Zornteufel“ („Baghira“) [21]; weitere Abklärung/Überweisung). Weiterhin konnten die FfPaed angeben, ob sie das Muster 36 ausgegeben haben, wie sie die Motivation der Familie zur Teilnahme an einem Präventionsprogramm einschätzen und, sofern die Familien eine Teilnahme am Präventionsprogramm sofort abgelehnt haben, welche Gründe aus ihrer Sicht dafür vorherrschend waren.

Der SDQ diente als Screeninginstrument zur Identifikation von Kindern mit psychischen Auffälligkeiten. Die Kinder wurden dabei von einem Elternteil bzgl. ihrer Stärken und Schwächen beurteilt. In der Version für 4‑ bis 17-Jährige werden mithilfe von 25 Items die 5 Skalen „Verhaltensprobleme“ (external), „Emotionale Probleme“ (internal), „Hyperaktivität“, „Probleme mit Gleichaltrigen“ und „Prosoziales Verhalten“ erfasst und können auf Ebene der Subskalen oder als Gesamtwert ausgewertet werden. Dabei gehen höhere Werte mit einer höheren Symptomlast einher.

Als „unauffällig“ wurden Kinder beurteilt, die einen Wert zwischen 0–3 (internal) bzw. 0–2 (external) erzielten. Um den Anteil der Kinder mit potenzieller Indikation einer Prävention möglichst auszuschöpfen, wurden die Cut-off-Werte für die SDQ-Subskalen „Emotionale Probleme“ und „Verhaltensprobleme“ insofern modifiziert, dass die Grenzwerte zum Bereich „hochauffällig“ um je 2 Punkte angehoben wurden (ursprünglich grenzwertig auffälliger Bereich [19]: Wert 4 auf der Skala „Emotionale Probleme“, Wert 3 auf der Skala „Verhaltensprobleme“; grenzwertig auffälliger Bereich im Rahmen dieser Studie: 4–6 (internal) bzw. 3–5 (external)). Familien, deren Kinder einen Wert im grenzwertig auffälligen Bereich zeigten, erhielten von ihrer FfPaed unter Berücksichtigung ihrer klinischen Expertise eine Präventionsempfehlung (oder ggf. eine alternative Empfehlung, z. B. klinische Abklärung). Für den Fall, dass die Werte auf beiden Subskalen im grenzwertig auffälligen Bereich lagen, wurde gemeinsam mit den Eltern im Rahmen des Vorgespräches abgewogen, von welchem Programm das Kind zunächst wahrscheinlich besser profitieren könnte. Sofern ein SDQ-Wert zwischen 7 und 10 (internal) bzw. 6 und 10 (external) vorlag, wurde eine nähere diagnostische Abklärung in ambulanten/stationären Einrichtungen empfohlen. Hierfür konnte die FfPaed den Familien eine Broschüre mit regionalen Ansprechpartnern übergeben (Abb. 1).

Das Fragebogenheft diente zur Erhebung von soziodemografischen Angaben der Familien sowie zur Erfassung von Barrieren und Förderfaktoren der Inanspruchnahme von Präventionsprogrammen aus Elternsicht.

Es wurden auch FfPaed und Familien befragt, die eine Studienteilnahme ablehnten oder im weiteren Verlauf am Vorgespräch oder an einem der Präventionsprogramme nicht teilnahmen. Im Fall, dass die FfPaed eine Studienteilnahme ablehnte, wurde nach vorheriger Einholung der Erlaubnis ein anonymisierter Arztfragebogen versendet. Zusätzlich wurde der Grund der Ablehnung mittels Antwortbogen schriftlich und bei Bedarf telefonisch erhoben. Bei unmittelbarer Ablehnung der Studienmappe von Familien erfragten die Praxismitarbeitenden mündlich die Gründe und dokumentierten diese auf dem sog. Dokumentationsbogen. Zudem wurden diese Familien gebeten, einen anonymen Nichtteilnehmer-Fragebogen vor Ort auszufüllen. Bei Familien, die sich nach dem Screening und einer Präventionsempfehlung nicht selbstständig beim Studienteam meldeten, wurde bei vorliegender Einwilligung zur Kontaktaufnahme versucht diese telefonisch, per E‑Mail oder postalisch zu kontaktieren. Falls Familien die Teilnahme am Vorgespräch oder nach abgeschlossenem Vorgespräch die Teilnahme an einem Präventionsprogramm ablehnten, wurden sie mündlich und mittels Online-Befragung nach den Gründen dazu befragt. Die FfPaed und die Familien konnten bei den Ablehnungsgründen Mehrfachangaben machen.

### Statistische Analysen

Die statistischen Analysen wurden mit STATA 17 [23] durchgeführt. Die Datenauswertung erfolgte mithilfe deskriptiver Statistiken (absolute/relative Häufigkeiten, Mittelwerte, Standardabweichungen). Für den Vergleich der Familien, die über teilnehmende Praxen vs. über andere Zugangswege am Projekt teilnahmen, erfolgten Tests auf Unabhängigkeit/Mittelwertsunterschiede mit Alpha a priori auf α = 0,05. Beim SDQ waren auf den Subskalen „Emotionale Probleme“/„Verhaltensprobleme“ maximal 2 fehlende Werte erlaubt, in diesen Fällen wurde eine personenbezogene Mittelwertimputation basierend auf dem Skalenmittelwert durchgeführt.

## Ergebnisse

### Teilnahmebereitschaft der FfPaed an der Umsetzung des Screenings

Von den kontaktierten FfPaed (*n* = 119) in Dresden und Umgebung nahmen 46 (38,7 %) aus insgesamt 28 Praxen am Projekt teil, dazu 5 angestellte Ärzt*innen. Von den FfPaed waren 37 weiblich und 9 männlich (Tab. 1). Das mittlere Alter lag bei 52 (Range: 36–65) Jahren. Eine Teilnahme an der Studie wurde von 47 (39,5 %) FfPaed abgelehnt (Tab. 3), 17 (14,3 %) gaben keine Rückmeldung und 9 (7,6 %) waren nicht kontaktierbar (z. B. Brief unzustellbar und telefonisch unerreichbar).

**Table 1:** 

	Teilnehmende FfPaed	Nicht-teilnehmende FfPaed^a^
Variable	(*n* = 46)	(*n* = 73)
**Alter,** M (SD) (*n)*]	51,5 (7,1) (38)	56,8 (13,2) (6)
**Geschlecht, ***n* (%)		
Weiblich	37 (80,4)	51 (69,9)
Männlich	9 (19,6)	22 (30,1)
**Art der Niederlassung, ***n* (%)		
Eigene Praxis	15 (32,6)	0 (0,0)
Gemeinschaftspraxis/Praxisgemeinschaft	31 (67,4)	1 (100,0)
**Niedergelassene Berufserfahrung in Jahren,** M (SD) (*n*)	13,0 (8,2) (39)	17,5 (11,3) (8)
**Zeit pro Patient in Minuten,** M (SD) (*n*)	11,6 (4,5) (35)	9,3 (3,4) (6)
**Durchschnittliche Anzahl Patienten pro Tag,** M (SD) (*n*)	46,2 (18,0) (32)	55,6 (20,6) (8)
**Anzahl U‑Untersuchungen pro Quartal,** M (SD) (*n*)	57,3 (52,5) (37)	57,5 (68,1) (7)
**Zeiten ohne U‑Untersuchungen,** *n* (%)		
Nein	36 (94,7)	7 (100,0)
Ja	2 (5,3)	0 (0,0)
**Anteil **(in %) **Patienten mit Migrationshintergrund,** M (SD) (*n*)	11,4 (9,7) (37)	7,8 (6,6) (6)
**Anzahl Mitarbeiter** (1 = Vollzeit), M (SD) (*n*)	3,7 (3,2) (37)	3,1 (1,1) (8)
**Einschätzung der Kompetenz hinsichtlich des Erkennens von psychischen Auffälligkeiten bei Kindern im Alter von 5–10 Jahren, ***n* (%)		
Schlecht	0	0
Eher schlecht	0	0
Mittelmäßig	11 (28,2)	3 (37,5)
Gut	26 (66,7)	2 (25)
Sehr gut	2 (5,1)	3 (37,5)
**Kenntnis von Präventionsprogrammen zur Förderung der seelischen Gesundheit,** *n* (%)		
Nein	25 (65,8)	7 (87,5)
Ja	13 (34,2)	1 (12,5)
**Kenntnis von Präventionsempfehlungsformular (Muster 36),** *n* (%)		
Nein	17 (46,0)	4 (50,0)
Ja	20 (54,0)	4 (50,0)
***Wenn ja:* Einsatz von Präventionsempfehlungsformular (Muster 36),** *n* (%)		
Nein	5 (25,0)	1 (25,0)
Ja	15 (75,0)	3 (75,0)
**Anzahl Weiterbildungsveranstaltungen zum Thema „psychische Erkrankungen“ in den letzten 2 Jahren, **M (SD) (*n*)	2,3 (1,9) (32)	1,2 (1,5) (8)
**Einsatz von Fragebögen zur Erhebung/Abklärung der psychischen Gesundheit, ***n* (%)		
Nein	12 (37,5)	6 (75,0)
Ja	20 (62,5)	2 (25,0)
***Wenn ja:* Häufigkeit des Einsatzes von Fragebögen pro Quartal,** M (SD) (*n*)	38,0 (46,5) (11)	k. A.
**Vorgehen, wenn erstmalig psychische Auffälligkeiten bei einem Patienten beobachtet werden ***(Mehrfachantwort mgl.), n* (%)		
Beratung	36 (92,3)	8 (100,0)
Überweisung an Psychologen/Psychotherapeuten	24 (61,5)	5 (62,5)
Abwarten	21 (53,8)	2 (25,0)
Überweisung an Psychiatrie	14 (35,9)	5 (62,5)
Krisenintervention	12 (30,8)	6 (75,0)
Einsatz Fragebogen/Checkliste	5 (12,8)	1 (12,5)
Medikation	0 (0,0)	1 (12,5)
Einsatz natürlicher/pflanzlicher Präparate	2 (5,1)	1 (12,5)
Selbst durchgeführte Psychotherapie	0 (0,0)	1 (12,5)
Überweisung an andere	3 (7,7)	1 (12,5)
Sonstiges	6 (15,4)	1 (12,5)

Missings sind durch teilweise ausgefüllte Fragebögen entstanden und die Prozentangaben beziehen sich auf die vorhandenen Daten

*k.* *A.* keine Angabe, *M* Mittelwert, *n* Anzahl Stichprobe, *SD* Standardabweichung

^a^„Nicht-teilnehmende FfPaed“ schließt ablehnende, keine Rückmeldung gebende und nicht kontaktierbare FfPaed ein

### Zugangswege in die Versorgung und Prävention

Vorrangig gelang Familien der Eintritt in die Versorgungskette über am Projekt teilnehmende Kinderarztpraxen. Insgesamt dokumentierten die Praxismitarbeitenden 3739 Anfragen zur Studienteilnahme an Familien, die ihr Kind zu einer U‑Untersuchung (U9–U11) vorstellten.

**Figure 2:**
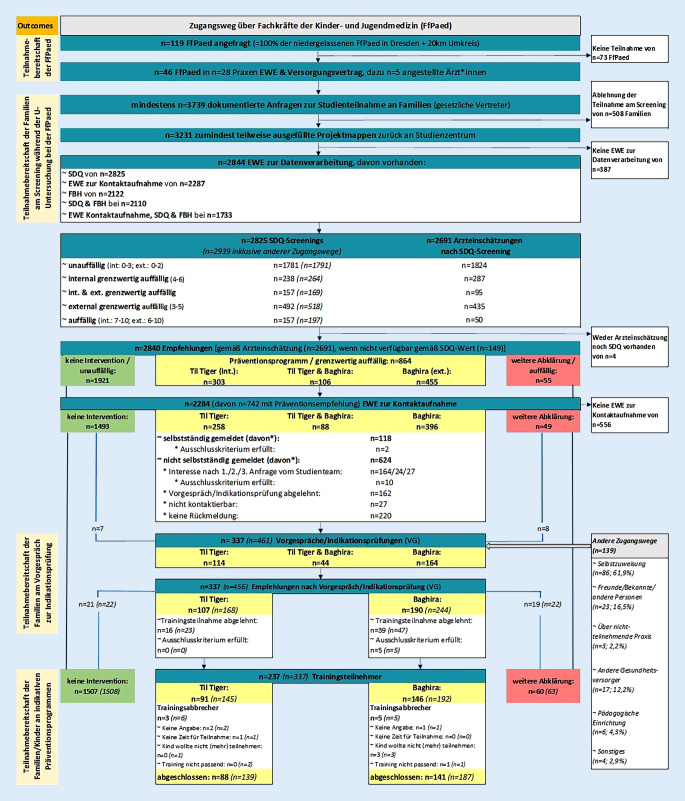

Zusätzlich fragten 139 Familien über andere Zugangswege eine Projektteilnahme an, davon nahmen 124 ein Vorgespräch wahr. 119/124 gaben anschließend ihre Einwilligung zur Datenverarbeitung (Abb. 2). Der Großteil (61,9 %) kam über Selbstzuweisung (z. B. Internetrecherche), gefolgt von Empfehlungen von Freunden/Bekannten/Anderen (16,5 %). Eine Stichprobenbeschreibung, getrennt nach Zugangswegen „über teilnehmende FfPaed“ und „andere“, findet sich in Tab. 2. Die Ergebnisse zeigen, dass Familien, die über andere Zugangswege in das Projekt kamen, häufiger Jungen vorstellten, häufiger SDQ-Ergebnisse im grenzwertig auffälligen/hochauffälligen Bereich erzielten, Väter zudem einen höheren Bildungsabschluss sowie Mütter weniger Wochenarbeitszeit angaben.

**Table 2:** 

	Über teilnehmende Praxis	Über andere Zugangswege	Vergleich	
Variable	(*n* = 2844^a^)	(*n* = 121^a^)	Chi^2^-/t-Test (df)	*p*-Wert
**Alter Kind, **M (SD) [*n*]	6,7 (2,0) [2823]	6,8 (1,7) [114]	−1,00 (126)	0,321
**Geschlecht Kind, ***n* (%)				
Weiblich	1413 (50,8)	31 (25,8)		
Männlich	1368 (49,2)	89 (74,2)	28,70 (1)	*<* 0,001*
**Nationalität Kind, ***n* (%)				
Deutsch	2045 (99,0)	51 (98,1)		
Andere	20 (1,0)	1 (1,9)	0,47 (1)	0,493
**U‑Untersuchung, ***n* (%)				
U9	1080 (52,2)			
U10	538 (26,0)			
U11	440 (21,3)			
Andere	11 (0,5)			n. b.
**Kind besucht derzeit:** *n* (%)				
Kindergarten/Vorschule	1087 (52,4)	24 (46,2)		
1. Klasse	127 (6,1)	10 (19,2)		
2. Klasse	305 (14,7)	7 (13,5)		
3. Klasse	252 (12,1)	8 (15,4)		
4. Klasse	192 (9,3)	2 (3,9)		
5. Klasse	112 (5,4)	1 (1,9)	17,18 (5)	0,004*
**Screening wurde ausgefüllt von:** *n* (%)				
Mutter	2233 (80,4)	92 (81,4)		
Vater	467 (16,8)	16 (14,2)		
Mutter und Vater	61 (2,2)	5 (4,4)		
Mutter und anderen	1 (0,0)	0 (0,0)		
Andere	16 (0,6)	0 (0,0)	3,53 (4)	0,474
**Kind lebt überwiegend bei: ***n* (%)				
Leiblicher Mutter	306 (14,7)	6 (11,5)		
Leiblichem Vater	21 (1,0)	0 (0,0)		
Beiden leiblichen Eltern	1684 (81,1)	44 (84,6)		
Leiblicher Mutter und anderen (z. B. Stiefvater)	49 (2,4)	1 (1,9)		
Leiblichem Vater und anderen (z. B. Stiefmutter)	3 (0,1)	1 (1,9)		
Pflege‑/Adoptiveltern	14 (0,7)	0 (0,0)	9,89 (5)	0,078
**Alter Sorgeberechtigter**^b^, M (SD) [*n*]				
Mutter	37,3 (4,8) [2057]	37,1 (5,3) [52]	0,42 (2107)	0,675
Vater	40,1 (5,9) [1935]	40,3 (5,8) [47]	−0,20 (1980)	0,843
**Nationalität Mutter**^b^, *n* (%)				
Deutsch	1995 (96,4)	50 (96,2)		
Andere	75 (3,6)	2 (3,8)	0,01 (1)	0,932
**Nationalität Vater**^b^, *n* (%)				
Deutsch	1881 (96,6)	45 (95,7)		
Andere	67 (3,4)	2 (4,3)	0,09 (1)	0,762
**Familienstand,** *n* (%)				
Alleinerziehend	282 (13,7)	6 (11,8)		
Verheiratet/feste Partnerschaft	1692 (82,0)	45 (88,2)		
Andere	90 (4,4)	0 (0,0)	2,59 (2)	0,274
**Arbeitszeit (Std./Woche), **M (SD) [*n*]				
Mutter^b^	31,7 (8,8) [1939]	27,9 (12,7) [51]	2,12 (51)	0,039*
Vater^b^	38,8 (6,5) [1853]	38,8 (4,8) [46]	0,12 (49)	0,904
**Nettoeinkommen Haushalt,** *n* (%)				
Unter 1000 €	26 (1,4)	0 (0,0)		
1000–2000 €	273 (15,1)	4 (8,7)		
2000–3000 €	345 (19,1)	11 (23,9)		
3000–4000 €	632 (35,0)	13 (28,3)		
Über 4000 €	530 (29,4)	18 (39,1)	−1,35 (1850)	0,178
**Höchster Bildungsabschluss Mutter**^b^, *n* (%)				
Ohne Abschluss	13 (0,6)	0 (0,0)		
Haupt‑/Volksschulabschluss	78 (3,8)	2 (3,9)		
Realschulabschluss/Mittlere Reife	698 (34,1)	13 (25,0)		
Abitur/Hochschulreife oder Fachhochschulreife	434 (21,2)	12 (23,1)		
Hochschulabschluss	791 (38,6)	25 (48,1)		
Anderes	35 (1,7)	0 (0,0)	−1,21 (2099)	0,228
**Höchster Bildungsabschluss Vater**^b^, *n* (%)				
Ohne Abschluss	15 (0,8)	0 (0,0)		
Haupt‑/Volksschulabschluss	109 (5,7)	4 (8,7)		
Realschulabschluss/Mittlere Reife	683 (35,5)	5 (10,9)		
Abitur/Hochschulreife oder Fachhochschulreife	317 (16,5)	9 (19,6)		
Hochschulabschluss	751 (39,1)	27 (58,7)		
Anderes	46 (2,4)	1 (2,2)	−2,53 (1965)	0,012*
**Ergebnisse aus SDQ**^c^, *n* (%)				
Unauffällig (int: 0–3; ext.: 0–2)	1781 (63,0)	10 (8,8)		
Internal grenzwertig (4–6)	238 (8,4)	26 (22,8)		
External grenzwertig (3–5)	492 (17,4)	26 (22,8)		
Internal und external grenzwertig	157 (5,6)	12 (10,5)		
Hochauffällig (int.: 7–10; ext.: 6–10)	157 (5,6)	40 (35,1)	−11,94 (120)	< 0,001*

*(df)* Freiheitsgrade, *ext.* external (Verhaltensprobleme), *M* Mittelwert, *int.* internal (emotionale Probleme), *n* Anzahl Stichprobe, *n.* *b.* nicht berechnet (z. B. aufgrund einer zu geringen Stichprobengröße), *SD* Standardabweichung

^a^Teilnahme am Screening mit Einwilligung zur Datenverarbeitung, Missings sind durch teilweise ausgefüllte Fragebögen entstanden und die Prozentangaben beziehen sich auf die vorhandenen Daten

^b^Mit „Mutter“ und „Vater“ sind die Personen gemeint, in deren Haushalt das Kind überwiegend lebt

^c^Insofern mindestens ein Wert auf den beiden relevanten SDQ-Subskalen im hochauffälligen Bereich war, wurde er dem hochauffälligen Bereich zugeordnet

*p < 0,05

### Teilnahmebereitschaft der Familien am Screening und an indikativen Präventionsmaßnahmen

3231/3739 (86,4 %) Familien beteiligten sich am Screening (Abb. 2); davon stimmten 2844/3231 (88,0 %) Familien der Datenverarbeitung zu.

Kinder, die am Screening teilnahmen, waren im Mittel 7 Jahre alt (Range: 4–11); darunter 1413 Mädchen und 1368 Jungen (keine Geschlechtsangabe von 63 Kindern). Das Screening wurde meist von den Müttern (*n* = 2233), seltener von den Vätern (*n* = 467) oder beiden leiblichen Elternteilen (*n* = 61) ausgefüllt (Tab. 2).

Insgesamt erhielten 2840 Familien eine Rückmeldung zu den SDQ-Ergebnissen im Rahmen einer U‑Untersuchung. Die Empfehlung basiert primär auf der Einschätzung der FfPaed („Arztbeurteilung“) nach dem SDQ-Screening (*n* = 2691); sofern keine Arztbeurteilung vorlag, basierte die Empfehlung ausschließlich auf dem SDQ-Wert (*n* = 149). Sobald die Einschätzung der FfPaed eine Präventionsempfehlung beinhaltete, wurde der Fall dem grenzwertig auffälligen Bereich (gelb in Abb. 1) zugeordnet. Sofern ein Wert mindestens auf einer der beiden relevanten SDQ-Subskalen im hochauffälligen Bereich (rot) lag, wurde er im hochauffälligen Bereich verortet.

864/2840 (30,4 %) teilnehmende Kinder erhielten eine Präventionsempfehlung. Davon kontaktierten 118/864 Familien (13,7 %) das Studienteam selbstständig. Von den übrigen 624 Familien mit Kontakteinwilligung wurden mehr als die Hälfte (*n* = 377, 60,4 %) vom Studienteam erreicht. 215/624 (34,5 %) meldeten auf Anfrage Interesse an einem Vorgespräch zurück, 162/624 (26,0 %) lehnten dieses ab. Die übrigen Familien konnten nicht kontaktiert werden (*n* = 27/624, 4,3 %; aufgrund fehlender/falscher Kontaktdaten) oder gaben keine Rückmeldung (*n* = 220/624, 35,3 %).

Mit 337 (39,0 %, *n* *=* *461*)^1^ Familien wurden Vorgespräche geführt, wovon 15 zwar keine Präventionsempfehlung erhielten, aber ein Vorgespräch wünschten. 107/337 (31,8 %, *n* *=* *168)* Familien erhielten von Studienmitarbeitenden nach dem Vorgespräch eine Empfehlung für das Programm „Mutig werden mit Til Tiger“ [20] und 190/337 (56,4 %, *n* = 244) für das „Baghira-Training“ [21]. Letztlich nahmen 91/107 (85,0 %, *n* = 145) Kinder an „Til Tiger“ und 146/190 (76,8 %, *n* = 192) an „Baghira“ teil. Das Tiger-Training haben 88/91 (96,7 %, *n* = 139) und das Baghira-Training 141/146 (96,6 %, *n* = 187) Kinder abgeschlossen. Ein Training abgebrochen haben 8/237 (3,4 %, *n* = 11) Kinder; die Gründe sind Abb. 2 zu entnehmen.

### Ablehnungsgründe

Seitens der FfPaed wurde die Ablehnung der Projektteilnahme hauptsächlich mit Zeitmangel (41,3 %) und „keine Durchführung von U‑Untersuchungen“ (19,1 %) begründet.

Eine Teilnahme am Screening lehnten die Eltern vorrangig aus fehlendem Interesse, Zeitmangel oder mangelnder(m) Notwendigkeit/Bedarf ab. Dieselben Ablehnungsgründe nannten die Familien nach Programmempfehlung und vor dem Vorgespräch, zusätzlich wurde eine zwischenzeitliche Problembesserung als Ablehnungsgrund angegeben. Als Hauptgründe für eine Ablehnung nach dem Vorgespräch bzw. vor Programmteilnahme führten Familien mangelnde(n) Notwendigkeit/Bedarf, ein unpassendes Programm und eine selbstständige Problembesserung an (Tab. 3).

**Table 3:** 

	Ablehnungsgründe *n*
**FfPaed**	
**Ablehnung der Projektteilnahme (*n*** **=** **47)**	Keine Zeit *n* = 19, keine U‑Untersuchungen *n* = 9, Patientenlast zu hoch *n* = 4, Personaldecke nicht ausreichend *n* = 4, Teilnahme an anderem Projekt *n* = 3, sieht keinen Nutzen im Projekt *n* = 1, andere *n* = 26
	*Andere: *Rente *n* = 7, Corona *n* = 4, keine Angabe *n* = 4, Umstrukturierung *n* = 3, arbeiten bereits therapeutisch *n* = 2, Distanz *n* = 2, Sonstiges *n* = 5
**Familien**	
**Ablehnung des *Screenings* aus Nichtteilnehmer-Fragebogen**^a^** (*n*** **=** **317)**	Keine Zeit *n* = 122, Sorge um Privatsphäre und Vertraulichkeit *n* = 44, kein Nutzen wird gesehen *n* = 30, Probleme verschwinden auch ohne Hilfe wieder *n* = 23, Probleme sollten innerhalb der Familie bleiben *n* = 14, Beeinträchtigung schulischer/beruflicher Zukunft *n* = 7, schlechte Vorerfahrungen gemacht *n* = 7, zu wenig Informationen über Projekt erhalten *n* = 5, Kind könnte dauerhaften Krankenakteneintrag bekommen *n* = 3, was könnten die anderen Leute bei Teilnahme denken *n* = 1, andere Gründe *n* = 159
	*Andere:* kein Bedarf/keine Notwendigkeit *n* = 97, kein Interesse *n* = 22, anderweitig angebunden *n* = 15, Kurzfristigkeit *n* = 5, fehlende Absprachemöglichkeiten z. B. mit anderen Eltern *n* = 5, Kind nicht in Zielgruppe *n* = 5, Kosten *n* = 3, schwere Organisation/hoher Aufwand *n* = 3, Programm nicht passend *n* = 1, verzogen *n* = 1, keine Angabe *n* = 1, Sonstiges *n* = 9
**Ablehnung der Teilnahme am *Screening* lt. Dokumentationsbogen**^b^** (*n*** **=** **508)**	Kein Interesse *n* = 131, keine Zeit *n* = 89, Kind privat versichert *n* = 28, Datenschutzbedenken *n* = 23, Projektnutzen fraglich *n* = 15, seelische Gesundheit des Kindes geht nur die Familie etwas an *n* = 10, schlechte Vorerfahrungen *n* = 7, andere Gründe *n* = 136
**Ablehnung nach Trainingsempfehlung und *vor Vorgespräch *aus der Datenbank**^c^** (*n*** **=** **162)**	Kein Bedarf/keine Notwendigkeit *n* = 76, keine Zeit *n* = 32, keine Angabe *n* = 25, kein Interesse *n* = 20, langer Anfahrtsweg *n* = 11, anderweitig angebunden *n* = 9, Programm nicht passend *n* = 6, schwere Organisation/hoher Aufwand *n* = 4, Corona *n* = 5, Verbesserung des Verhaltens *n* = 3, verzogen *n* = 2, Sonstiges *n* = 7
**Ablehnung nach Trainingsempfehlung und *vor Vorgespräch* aus dem Nichtteilnehmer-Fragebogen**^d^** (*n*** **=** **61)**	Problem wird als nicht so ernsthaft eingeschätzt *n* = 24, Problem hat sich selbstständig gebessert *n* = 22, keine Zeit für Teilnahme *n* = 19, finde das empfohlene Programm unpassend für mein Kind *n* = 13, Einrichtung ist schwer zu erreichen *n* = 7, Kind möchte nicht teilnehmen *n* = 5, Sorgen um Vertraulichkeit und die Privatsphäre *n* = 2, mein Kind und ich fühlen uns fehl am Platz *n* = 2, Sonstiges *n* = 17
	*Andere*: Anfahrt zu lange *n* = 5, keine geeigneten Parkmöglichkeiten *n* = 1, keine angemessene Verbindung zum öffentlichen Nahverkehr *n* = 1
**Ablehnung nach dem Vorgespräch und *vor Trainingsteilnahme *aus der Datenbank**^c^ ***n*** **=** **55 *(n*** ***=*** ***70)***	Keine Angabe *n* = 12 *(n* *=* *18)*, kein Bedarf/keine Notwendigkeit *n* = 7 *(n* *=* *11)*, keine Rückmeldung *n* = 9 *(n* *=* *9)*, Programm nicht passend *n* = 7 *(n* *=* *7)*, keine Zeit *n* = 4 *(n* *=* *5)*, kein Interesse *n* = 2 *(n* *=* *2)*, anderweitig angebunden *n* = 2 *(n* *=* *6)*, langer Anfahrtsweg *n* = 5 *(n* *=* *6)*, schwere Organisation/hoher Aufwand *n* = 2 *(n* *=* *2)*, verzogen *n* = 2 *(n* *=* *2)*, Kind möchte nicht teilnehmen *n* = 3 *(n* *=* *3)*, Krankenkasse übernimmt Kosten nicht *n* = 1 *(n* *=* *1)*, Fragebogen vom Studienteam nicht versandt *n* = 1 *(n* *=* *1)*, Sonstiges *n* = 2 *(n* *=* *3)*
**Ablehnung nach dem Vorgespräch und *vor Trainingsteilnahme* aus dem Nichtteilnehmer-Fragebogen**^d^ ***n*** **=** **18 *(n*** ***=*** ***27)***	Problem hat sich selbstständig gebessert *n* = 6 *(n* *=* *9)*, keine Zeit für Teilnahme *n* = 5 *(n* *=* *8)*, Kind möchte nicht teilnehmen *n* = 5 *(n* *=* *7), *finde das empfohlene Programm unpassend für mein Kind *n* = 5 *(n* *=* *5)*, Problem wird als nicht so ernsthaft eingeschätzt *n* = 3 *(n* *=* *4), *mein Kind und ich fühlen uns fehl am Platz *n* = 2 *(n* *=* *3)*, die Einrichtung ist schwer zu erreichen *n* = 1 *(n* *=* *3, *davon: lange Anfahrtszeit *n* = 1 *(n* *=* *3))*, Probleme mit der Krankenkasse *n* = 1 *(n* *=* *1, *davon: Krankenkasse übernimmt Kosten nicht oder nur teilweise *n* = 1 *(n* *=* *1)*, Gutscheinverfahren zu umständlich *n* = 1 *(n* *=* *1))*, Zeitraum bis Trainingsbeginn zu lange *n* = 0 *(n* *=* *2), *Sonstiges *n* = 5 *(n* *=* *9)*

*(kursiv)* = Anzahl gesamt (Zugang über FfPaed in teilnehmenden Praxen und andere Zugangswege), *n* Stichprobenanzahl

^a^Wurde von Praxispersonal an Familie ausgegeben, wenn diese dazu bereit war, ihn auszufüllen

^b^Darauf wurden die Ablehnungsgründe nach mündlicher Nachfrage vom Praxispersonal vermerkt

^c^Datenerhebung meist mündlich über Telefon

^d^Datenerhebung mithilfe eines Online-Fragebogens

## Diskussion

In vorliegender Studie wurden die Umsetzbarkeit und Akzeptanz einer innovativen Versorgungskette untersucht, die den Zugang zu indikativen Präventionsmaßnahmen für Kinder mit erhöhtem Risiko für psychische Störungen erleichtern und deren Inanspruchnahme erhöhen sollte.

Die Ergebnisse lassen darauf schließen, dass die Ausweitung der regulären U‑Untersuchungen auf psychische Auffälligkeiten seitens der FfPaed und Familien gut angenommen wurde. Die Beteiligung der FfPaed (38,7 %) und der Familien am Screening war akzeptabel, gleichwohl fiel die Ablehnungsrate der Familien mit 13,6 % deutlich höher aus als erwartet (Pilotprojekt: 5 %). Die höhere Ablehnungsrate lag womöglich darin begründet, dass im Pilotprojekt nur der SDQ im Gegensatz zu einer umfangreicheren Mappe, wie im PROMPt-Projekt, an die Familien ausgegeben wurde und die Angaben vollständig anonym erhoben wurden. Als Hauptgrund für die Ablehnung der Teilnahme am Projekt (FfPaed)/am Screening (Familien) wurde Zeitmangel genannt. Bedenkt man, dass bei einer Implementierung des Screenings in die Regelversorgung, im Gegensatz zum Projektvorgehen, zeitintensive Formulare (Studieninformation/EWE/Fragebogenheft) entfallen und einzig der SDQ ausgefüllt würde, ist eine höhere Teilnahmequote wahrscheinlich. Ebenso ist durch den in der Praxis überschaubaren und zudem geteilten Mehraufwand (Ausgabe/Auswertung übernimmt Praxispersonal; Rückmeldung leistet die FfPaed) die Durchführung in der Routine denkbar. Diese Argumentation wird durch qualitative Analysen gestützt [22].

Basierend auf dem SDQ-Screening erhielten 30,4 % der Kinder eine Präventionsempfehlung. Im Vergleich zu anderen Studien ist die Häufigkeit grenzwertig Auffälliger deutlich erhöht [24–26]. Zwar hat durch die COVID-19-Pandemie die Anzahl psychischer Belastungen durch soziale Isolation, Unsicherheiten und vermehrte Familienkonflikte deutlich zugenommen [27]. Ausschlaggebender Faktor scheint jedoch eine leichte projektbedingte Modifikation der Cut-off-Werte zu sein, sodass weniger Kinder als „hochauffällig“ bzw. häufiger als „grenzwertig auffällig“ klassifiziert wurden.

Erfreulicherweise gab es außerhalb der Zuweisung über die FfPaed auch Familien, die vornehmlich selbstinitiativ auf die Präventionsangebote aufmerksam wurden und Interesse an einer Programmteilnahme angaben. Dies zeigt, dass Eltern psychische Auffälligkeiten ihrer Kinder wahrnehmen und Unterstützung suchen.

Bedenklich war, dass sich nur wenige Familien (*n* = 118/864) nach erhaltener Präventionsempfehlung selbstständig bei Studienmitarbeitenden für das Präventionsprogramm meldeten, wenngleich bei aktiver Kontaktaufnahme seitens des Studienteams mehr als ein Drittel (34,5 %) der Familien das Vorgespräch wahrnahm. Diese Erkenntnis untermauert den Befund [28], dass für Familien mit Bedarfen Angebote zur Prävention stärker bekanntgemacht und beworben werden müssen, um eine Inanspruchnahme herbeizuführen. Diese Argumentation wird unterstützt durch den Befund, dass Familien, die im Rahmen des Vorgespräches eine Präventionsempfehlung erhielten, in der Mehrzahl der Fälle (82,5 % über alle Zugangswege hinweg) das Präventionsangebot in Anspruch nahmen und dieses selten abbrachen. Die geringe Abbruchrate für die Teilnahme an den Präventionsprogrammen (3,3 % über alle Zugangswege hinweg) ist womöglich auf das Vorgespräch zurückzuführen. Ein solches kann wie in dieser Studie der Indikationsprüfung, Aufklärung über Inhalt und Rahmenbedingungen der Programme dienen, die Teilnahmemotivation der Familie und des Kindes zu eruieren sowie das Einverständnis des Kindes für die Teilnahme einzuholen. Es muss auch davon ausgegangen werden, dass eine klare, strukturierte und die nächsten konkreten Schritte benennende Empfehlung der Vertrauensperson (FfPaed), die auch weiterhin von den Familien aufgesucht wird, zur hohen Haltequote geführt hat. Durch dieses Vorgehen wurde die teils schwierige „Brücke“ von zunächst somatisch-orientierter Betrachtung hin zu psychosozialen Aspekten kindlicher Entwicklung neben der Nennung von Ansprechpersonen und Angeboten klar und haltend gemeistert. Diese Aspekte trugen vermutlich maßgeblich zu einer informierten und partizipativen Entscheidung für die Teilnahme an einem Präventionsprogramm und damit auch zu den hohen Haltequoten bei.

Ergänzend zeigten die Untersuchungen, dass Prävention psychischer Auffälligkeiten im derzeitigen Versorgungssystem bei den FfPaed eher eine untergeordnete Rolle spielt. So setzten zwar 62,5 % der teilnehmenden FfPaed im Durchschnitt 38 Mal pro Quartal ein Screeninginstrument zur Erfassung psychischer Auffälligkeiten ein, allerdings wurden keine Präventionsprogramme empfohlen, sondern eher beraten, abgewartet („watchful waiting“) oder an Psychotherapeuten/Psychologen überwiesen (Tab. 1). Diese Ergebnisse spiegeln möglicherweise Resultate vorangegangener Untersuchungen wider, welche Schwierigkeiten bei der Beratung und Weitervermittlung offenlegten [14]. Mit Ausnahme dieses Studienprogrammes werden indikative Präventionsangebote nicht regelhaft vorgehalten; damit ist für FfPaed häufig unklar, wohin sie im Falle einer identifizierten und persistierenden psychischen Auffälligkeit vermitteln können. Wichtig ist daher, dass entsprechende regionale Versorgungsangebote verfügbar und bekannt sind.

Kurative Maßnahmen und allgemein gesundheitsförderliche Maßnahmen sollten durch gezielte primär- und sekundärpräventive Maßnahmen erweitert werden. Der Zwischenbereich der indikativen Prävention ist allerdings im Hinblick auf Gesetz und Struktur unzureichend ausgebaut und für Programmanbieter aus ökonomischer Sicht wenig reizvoll. Vergleicht man die Teilnahmegebühren für ein gesamtes Präventionsprogramm (130 € pro Kind, 4–6 Teilnehmende mgl., 11 Sitzungen à 60 min; [20]) mit einer psychotherapeutischen Behandlung (103,87 € für Einzelsitzung à 50 min oder 339,28 € für Gruppensitzung mit 4 Teilnehmenden à 100 min; Vergütung laut EBM-Katalog der Kassenärztlichen Bundesvereinigung) und berücksichtigt, dass dringend benötigte Therapieplätze in ungenügendem Ausmaß vorhanden und mit langen Wartezeiten verbunden sind [27], scheint ein Paradigmenwechsel hin zu einer stärker auf indikative Prävention ausgerichteten Versorgung äußerst lohnenswert.

### Limitationen

Aufgrund der Datenerhebung mittels Fragebogen war es Familien mit unzureichenden Deutschkenntnissen/Lesefertigkeiten nur schwer möglich, an der Befragung teilzunehmen. Der Anteil von Kindern und Eltern mit Migrationshintergrund (Tab. 2) liegt zudem deutlich unter dem erwarteten Anteil für Dresden (13,8 %, Stand 12/2021) [29]. Die Versorgungskette ist ohne Berücksichtigung regionaler Versorgungsstrukturen/-angebote sowie der Versorgungsdichte [30, 31] nicht ohne Weiteres übertragbar, da regionale Unterschiede in den Angebotsstrukturen zur Prävention, Beratung und Behandlung sowie bei der Ärzteschaft bestehen. Damit wäre die Übertragbarkeit der Ergebnisse dennoch nicht per se eingeschränkt; allerdings könnten in ländlichen Räumen strukturelle Barrieren, z. B. Anfahrtswege und Erreichbarkeit/Verfügbarkeit von Präventionsangeboten, die Inanspruchnahme stärker als in der vorliegenden Studie beeinflussen.

## Fazit

Die Untersuchungen zeigten, dass die erprobte Versorgungskette mit Beginn des Screenings psychischer Auffälligkeiten in der Kinderarztpraxis bis hin zur Inanspruchnahme eines indikativen Präventionsprogrammes in die Regelversorgung implementierbar und umsetzbar ist sowie Akzeptanz (i. S. einer Teilnahmebereitschaft) aufseiten der FfPaed und Familien erfährt.

Um die Teilnehmerraten an Präventionsprogrammen zu erhöhen, muss Prävention aber auch aktiv gefördert werden. Aufklärungskampagnen mithilfe zielgruppenspezifischer Kommunikationsstrategien (z. B. Ausgabe von Flyern in Kitas/Schulen/Vereinen/Beratungsstellen; Informationsvermittlung über soziale Medien) und Anreize (z. B. kostenfreie Programmteilnahme) könnten dabei unterstützen. Die wohl größte Herausforderung ist die Herstellung einer nachhaltigen Versorgungsstruktur, in der sich einerseits FfPaed mühelos orientieren und die Familien an passgenaue Hilfen weitervermittelt werden können. Andererseits müssen evidenzbasierte indikative Präventionsangebote flächendeckend angeboten werden. Eine vielversprechende Möglichkeit für die Umsetzung stellt die Implementierung von indikativen Präventivmaßnahmen in den Lebenswelten (Schule/Kindergarten/Hort/Vereine) der Kinder dar. Der niederschwellige Zugang zu den Interventionen könnte dadurch erleichtert werden und nachhaltig zu einer Entstigmatisierung im Umgang mit psychischen Beschwerden und zur Erreichbarkeit unterschiedlicher Zielgruppen und damit einhergehender Minderung gesundheitlicher Ungleichheit beitragen sowie im weitesten Sinne Folgekosten vermeiden und die Lebenseinstiegschancen erhöhen.

## Footnotes

1 *Anzahl gesamt (Zugang über FfPaed in teilnehmenden Praxen und über andere Wege).*
